# Trends in Worldwide Research in Inflammatory Bowel Disease Over the Period 2012–2021: A Bibliometric Study

**DOI:** 10.3389/fmed.2022.880553

**Published:** 2022-05-19

**Authors:** Kemin Li, Chenzhe Feng, Haolin Chen, Yeqian Feng, Jingnan Li

**Affiliations:** ^1^Department of Gastroenterology, Peking Union Medical College, Peking Union Medical College Hospital, Chinese Academy of Medical Sciences, Beijing, China; ^2^Key Laboratory of Gut Microbiota Translational Medicine Research, Peking Union Medical College, Peking Union Medical College Hospital, Chinese Academy of Medical Sciences, Beijing, China; ^3^Department of Oncology, Second Xiangya Hospital of Central South University, Changsha, China; ^4^Department of Surgery, Xiangya Hospital of Central South University, Changsha, China; ^5^Department of Mathematics, University of California, Davis, Davis, CA, United States

**Keywords:** inflammatory bowel disease, bibliometric, machine learning, publication analysis, coronavirus disease 2019

## Abstract

**Background:**

Inflammatory bowel disease (IBD) is a continuously increasing and worldwide disease, and the number of publications of IBD has been expanding in the past 10 years. The purpose of this study is to analyze the published articles of IBD in the past decade via machine learning and text analysis and get a more comprehensive understanding of the research trends and changes in IBD in the past 10 years.

**Method:**

In November 2021, we downloaded the published articles related to IBD in PubMed for the past 10 years (2012–2021). We utilized Python to extract the title, publication date, MeSH terms, and abstract from the metadata of each publication for bibliometric assessment. Latent Dirichlet allocation (LDA) was used to the abstracts to identify publications' research topics with greater specificity.

**Result:**

We finally identified and analyzed 34,458 publications in total. We found that publications in the last 10 years were mainly focused on treatment and mechanism. Among them, publications on biological agents and Gastrointestinal Microbiome have a significant advantage in terms of volume and rate of publications. In addition, publications related to IBD and coronavirus disease 2019 (COVID-19) have increased sharply since the outbreak of the worldwide pandemic caused by novel β-coronavirus in 2019. However, researchers seem to pay less attention to the nutritional and psychological status of patients with IBD.

**Conclusion:**

IBD is still a worldwide disease of concern with the publication of IBD-related research has expanded continuously over the past decade. More research related nutritional and psychological status of patients with IBD is needed in the future. Besides, it is worth noting that the management of chronic diseases such as IBD required additional attention during an infectious disease epidemic.

## Introduction

Inflammatory bowel disease (IBD), including ulcerative colitis (UC) and Crohn's disease (CD), is a nonspecific chronic inflammatory condition that affects the intestine (1). The incidence has been increasing consistently over the past decades, especially in newly industrialized countries (2), suggesting IBD is a global disease. In China, the incidence of UC has increased nearly threefold in recent years (3). Nearly half of IBD patients require hospitalization during the disease course, and 10–20% of patients undergo surgery during follow-up (4–6). Due to its chronic recurrent nature, the acute phase severely affects the life treatment and work efficiency of IBD patients. In addition, nearly 1.5% of UC patients develop colon cancer after 15 years of follow-up (4). However, the pathogenesis is still unclear, and it is thought to develop through complicated interactions between environmental, microbial, immune and genetic factors (7). The evolution of IBD can be divided into 4 stages: emergence, acceleration in incidence, compounding prevalence and prevalence equilibrium. Different stages will face different problems and challenges (8). Currently, the main treatment strategies are classified as step-up and top-down, and with the emergence of an increasing number of immunosuppressive and biological agents, the rational choice of treatment remains an issue of concern (9). Therefore, the pathogenesis, diagnosis and treatment of the disease still require further research.

Understanding the current research in the area of IBD helps us to grasp research in the present and points to the directions of our future research. Bibliometrics, using mathematical and statistical methods, are often referred to quantitatively analyze academic literature. Currently, bibliometric methods have been used to investigate the field of IBD, but all these studies have some shortcomings. For example, Chen et al. analyzed only the 100 most cited articles on IBD (10). Chong et al. performed an analysis only for IBD stem cell therapy (11). Weintraub et al. showed an analysis of the literature on IBD from 1993 to 2011 (12). Remarkably, the panorama of IBD-related research in the last ten years is ill-defined.

Natural language processing (NLP) is a computational technique utilized to analyze bibliometric research (13), and it has been effectively used to analyze research profiles in the field of cancer rehabilitation and glioma (14, 15). Our study aims to conduct a comprehensive exploration of the literature on IBD in the last 10 years based on the PubMed database via data analysis and natural language processing methods. This paper points out the hotspots in previous and current studies, presents future research opportunities for researchers and funding agencies, and is aware of the current research bias.

## Method

The methodology used in this study continues to follow previous studies (14, 15). We downloaded all publications indexed under the Mesh term “Inflammatory Bowel Disease.” From the public version of PubMed in November 2021, limited by publication data from 2012 to 2021. The full record of the search results was downloaded in XML format, extracting metadata from the original XML file. The extracted metadata included the title, abstract, keywords, MeSH words and year of publication for each article.

To identify the research topics in each article more specifically, we used the most classic topic modeling method (16, 17), named latent Dirichlet allocation (LDA), to distinguish discrete topics in a large number of unstructured texts and create its own feature glossary based on the frequency of terms appearing in a document set. Specifically, in the LDA, each topic is modeled as an infinite mixture of a set of base topic probabilities. Once the glossary was invented, the topic with the highest probability is defined as the main topic of each article by analyzing the abstract of the article based on the topic probability calculated by the algorithm.

In our study, we set the number of identified topics to 30, and the process refers to Supplementary Material. By analyzing the article abstracts, the topic with the highest probability calculated by the algorithm was defined as the main topic of each article. We finally performed 30 topics by manually checking based on abstract and MeSH terms and combining them with article abstracts. Then, we used the Louvain algorithm for cluster analysis and building a topic network so that we could find the relationships between topics and establish communities of related topics. For each article, we noted the two topics with the highest probability of their attribution, counted the frequency of each topic in each document, and linked the topics.

All relevant Python and R language codes can be found in the cited literature, and the original publication dataset was uploaded and stored to Zenodo (https://doi.org/10.5281/zenodo.6419975). All descriptive statistics are reported as the mean ± standard deviation. The network visualization in the article was performed using Excel and Gephi (https://gephi.org/). This article is a bibliometric analysis and does not require institutional review board or ethics committee approval.

## Result

**Figure 1:**
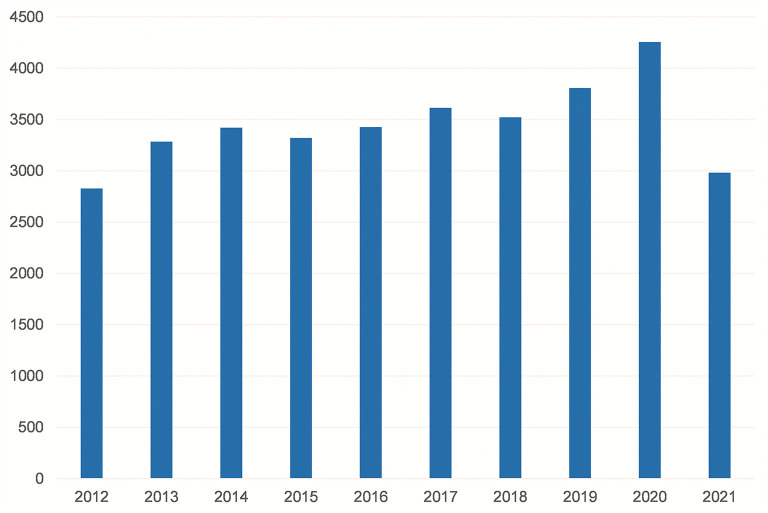
PubMed search results: articles per year.

The search reported 34,458 publications in total, including 30,178 Journal Articles, 6,904 Reviews, 3,558 Case Reports and 393 Clinical Trials. As shown in Figure 1, the number of publications per year from 2012 to 2021 showed a slow upward trend, with an average of 3,445.8 research papers related to IBD published per year. We found a decrease in the number of articles published in 2021, which may be related to the delay in indexing on the one hand and to the fact that 2021 is not yet over, rather than the decrease in the number of ongoing studies or publications.

## MeSH Analysis

We removed disease-specific words (e.g., inflammatory bowel disease, ulcerative colitis, Crohn's disease) and instrument-specific words (such as Magnetic Resonance Imaging, Immunohistochemistry) from the MeSH terms.

**Figure 2:**
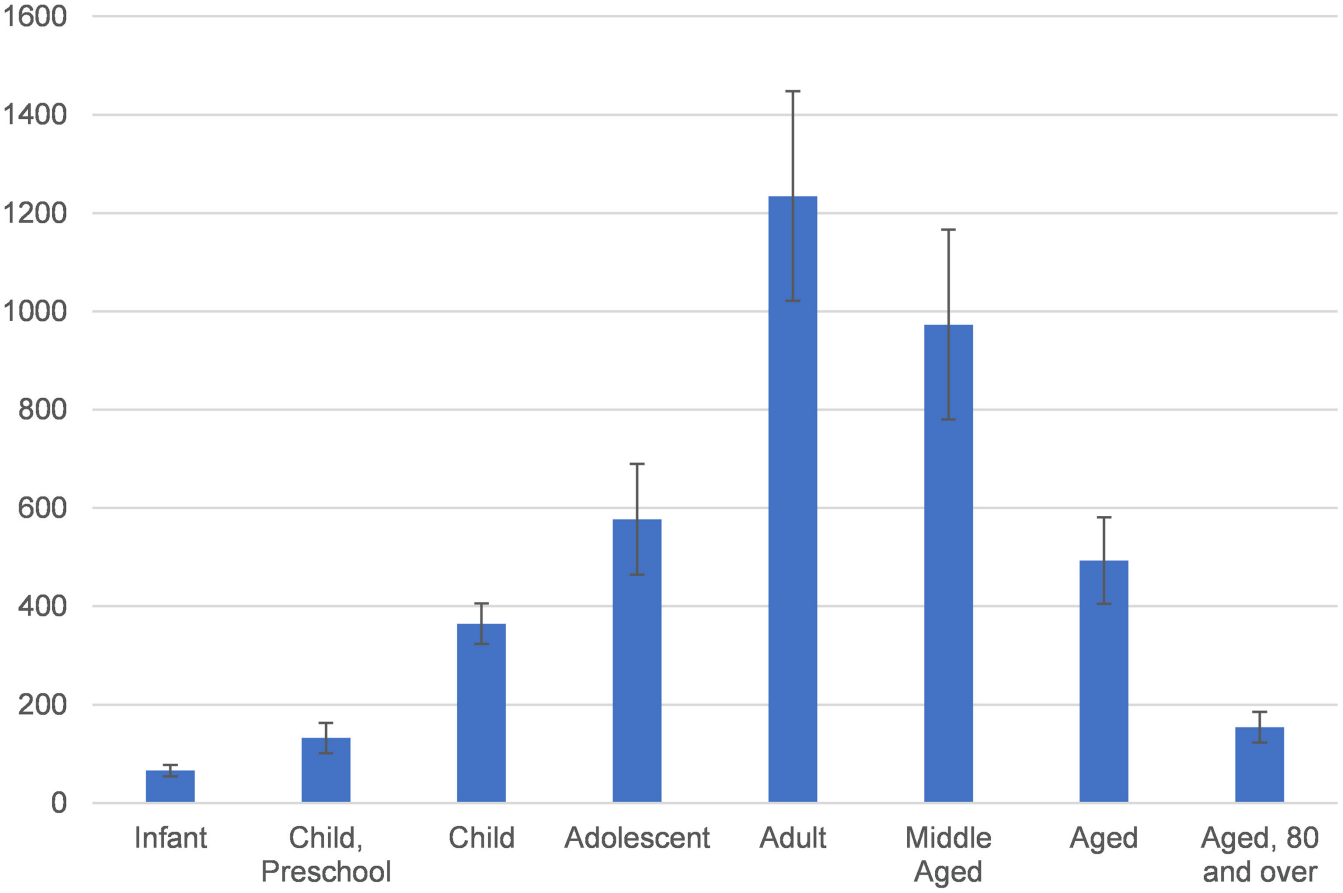
Annual publication of IBD-related literature, divided by age group.

Table 1 shows the top 20 MeSH terms that appeared in the retrieved articles that were thematically relevant. The most frequently occurring terms include T*reatment Outcome, Intestinal Mucosa, Risk Factors, Tumor Necrosis Factor-alpha*, and *Infliximab*. Figure 2 exhibits the annual relevant publications coverage for different age groups over the last 10 years. We divided eight age groups: infants (2–3 months of age), preschoolers (2–5 years), children (6–12 years), adolescents (13–18 years), adults (19–44 years), middle-aged adults (45–64 years), aged adults (65–80 years) and older adults (80 years and above). Articles dealing with more than one age group are included in the total number of publications for all corresponding age groups. Overall, the adult and middle-aged groups hold the most prominent status among the publications covered in this study, followed by the adolescent group.

**Table 1:** Overall ranking of research foci in the past 10 years.

**Rank**	**MeSH term**	**Record of occurrence in publications**
1	Treatment outcome	4,410
2	Intestinal mucosa	3,562
3	Risk factors	3,142
4	Tumor necrosis factor-alpha	2,967
5	Infliximab	2,770
6	Severity of illness index	2,713
7	Mice	2,704
8	Gastrointestinal agents	2,273
9	Immunosuppressive agents	2,228
10	Biomarkers	2,197
11	Anti-inflammatory agents	2,157
12	Disease models, animal	1,858
13	Inflammation	1,837
14	Prognosis	1,799
15	Antibodies, monoclonal	1,783
16	Colonoscopy	1,741
17	Feces	1,573
18	Gastrointestinal microbiome	1,477
19	Time factors	1,393
20	Adalimumab	1,389

We conducted a literature publication subject term analysis of the MeSH terms of publications every 2 years for the 10 years from 2012-2021.

Table 2 shows the top 15 popular terms every 2 years during the last 10 years. We noticed that *Treatment Outcome, Intestinal Mucosa, Risk Factors, Tumor Necrosis Factor-alpha, Infliximab, Anti-Inflammatory Agents, Mice*, and *Biomarkers* consistently appear in the top 15 research hotspots in the past decade. *Treatment Outcome, Intestinal Mucosa* and *Risk Factors* were ranked in the top 5. *Antibodies, Monoclonal, Immunosuppressive Agents* and *Prognosis* decrease people interest gradually until they disappeared in the last 2 years. In addition, *Gastrointestinal Agents, Gastrointestinal Microbiome, Disease Models, Animal*, and *Inflammation* have attracted huge attention in the past 6 years, while it is worth noting that *Gastrointestinal Microbiome* has been soaring up to the top 5 topics these 2 years. Some topics that appear in the top 15 in only one 2-year period, such as *Time Factors, Genetic Predisposition to Disease, Colonoscopy, Feces, Remission Induction, Anti-Inflammatory Agents, Non-Steroidal, COVID-19*, and *SARS-CoV-2*. Notably, the number of articles on the themes of *COVID-19* and *SARS-CoV-2* appeared since 2019 and have increased dramatically over the past 2 years.

**Table 2:** Top 15 research foci in each 2-years period examined.

**Rank**	**2012–2013**	**2014–2015**	**2016–2017**	**2018–2019**	**2020–2021**
1	Treatment outcome	Animals	Treatment outcome	Treatment outcome	Treatment outcome
2	Antibodies, monoclonal	Treatment outcome	Intestinal mucosa	Intestinal mucosa	Intestinal mucosa
3	Intestinal mucosa	Risk factors	Risk factors	Mice	Mice
4	Risk factors	Intestinal mucosa	Tumor necrosis factor-alpha	Tumor necrosis factor-alpha	Gastrointestinal microbiome
5	Immunosuppressive agents	Tumor necrosis factor-alpha	Infliximab	Risk factors	Infliximab
6	Tumor necrosis factor-alpha	Severity of illness index	Severity of illness index	Gastrointestinal agents	Risk factors
7	Infliximab	Immunosuppressive agents	Gastrointestinal agents	Severity of illness index	Severity of illness index
8	Anti-inflammatory agents	Infliximab	Mice	Infliximab	Disease models, animal
9	Severity of illness index	Antibodies, monoclonal	Biomarkers	Biomarkers	Inflammation
10	Prognosis	Anti-inflammatory agents	Immunosuppressive agents	Disease models, animal	Tumor necrosis factor-alpha
11	Mice	Prognosis	Anti-inflammatory agents	Gastrointestinal microbiome	Biomarkers
12	Anti-inflammatory agents, non-steroidal	Gastrointestinal agents	Colonoscopy	Inflammation	COVID-19
13	Biomarkers	Mice	Gastrointestinal microbiome	Anti-inflammatory agents	Gastrointestinal agents
14	Antibodies, monoclonal, humanized	Biomarkers	Feces	Prognosis	Anti-inflammatory agents
15	Genetic predisposition to disease	Time factors	Remission induction	Immunosuppressive agents	SARS-CoV-2

Similarly, we ranked the MeSH terms related to the treatment of IBD in the literature over time (Table 3). Articles on Immunosuppressive Agents, Gastrointestinal Agents, Infliximab, Anti-Inflammatory Agents, Remission Induction and Adalimumab appear among the top 10 topic in every 2-year period. Among them, Infliximab and Anti-Inflammatory Agents have been ranked in the top 5, meanwhile Gastrointestinal Agents and Remission Induction have gained more and more attention. Besides, the Mesh term of Postoperative Complications is almost always a hot topic of concern. In addition, the topics of Anti-Inflammatory Agents and Non-Steroidal were of high concern for the first 4 years and then disappeared out of the top 10. In turn, literature on the theme of colectomy appeared in the top 10 hotspots in the past 8 years. Of interest, we found that the number of publications with MeSH terms of Tumor Necrosis Factor Inhibitors jumped to the top 10.

**Table 3:** Top 10 research foci related to treatment in each 2-years period.

**Rank**	**2012–2013**	**2014–2015**	**2016–2017**	**2018–2019**	**2020–2021**
1	Antibodies, monoclonal	Immunosuppressive agents	Infliximab	Gastrointestinal agents	Infliximab
2	Immunosuppressive agents	Infliximab	Gastrointestinal agents	Infliximab	Gastrointestinal agents
3	Infliximab	Antibodies, monoclonal	Immunosuppressive agents	Anti-inflammatory agents	Anti-inflammatory agents
4	Anti-inflammatory agents	Anti-inflammatory agents	Anti-inflammatory agents	Immunosuppressive agents	Remission induction
5	Anti-inflammatory agents, non-steroidal	Gastrointestinal agents	Remission induction	Remission induction	Tumor necrosis factor inhibitors
6	Antibodies, monoclonal, humanized	Anti-inflammatory agents, non-steroidal	Adalimumab	Antibodies, monoclonal, humanized	Immunosuppressive agents
7	Gastrointestinal agents	Remission induction	Colectomy	Adalimumab	Adalimumab
8	Remission induction	Antibodies, monoclonal, humanized	Antibodies, monoclonal	Colectomy	Antibodies, monoclonal, humanized
9	Postoperative complications	Adalimumab	Postoperative complications	Postoperative complications	Postoperative complications
10	Adalimumab	Colectomy	Antibodies, monoclonal, humanized	Antibodies, monoclonal	Colectomy

**Figure 3:**
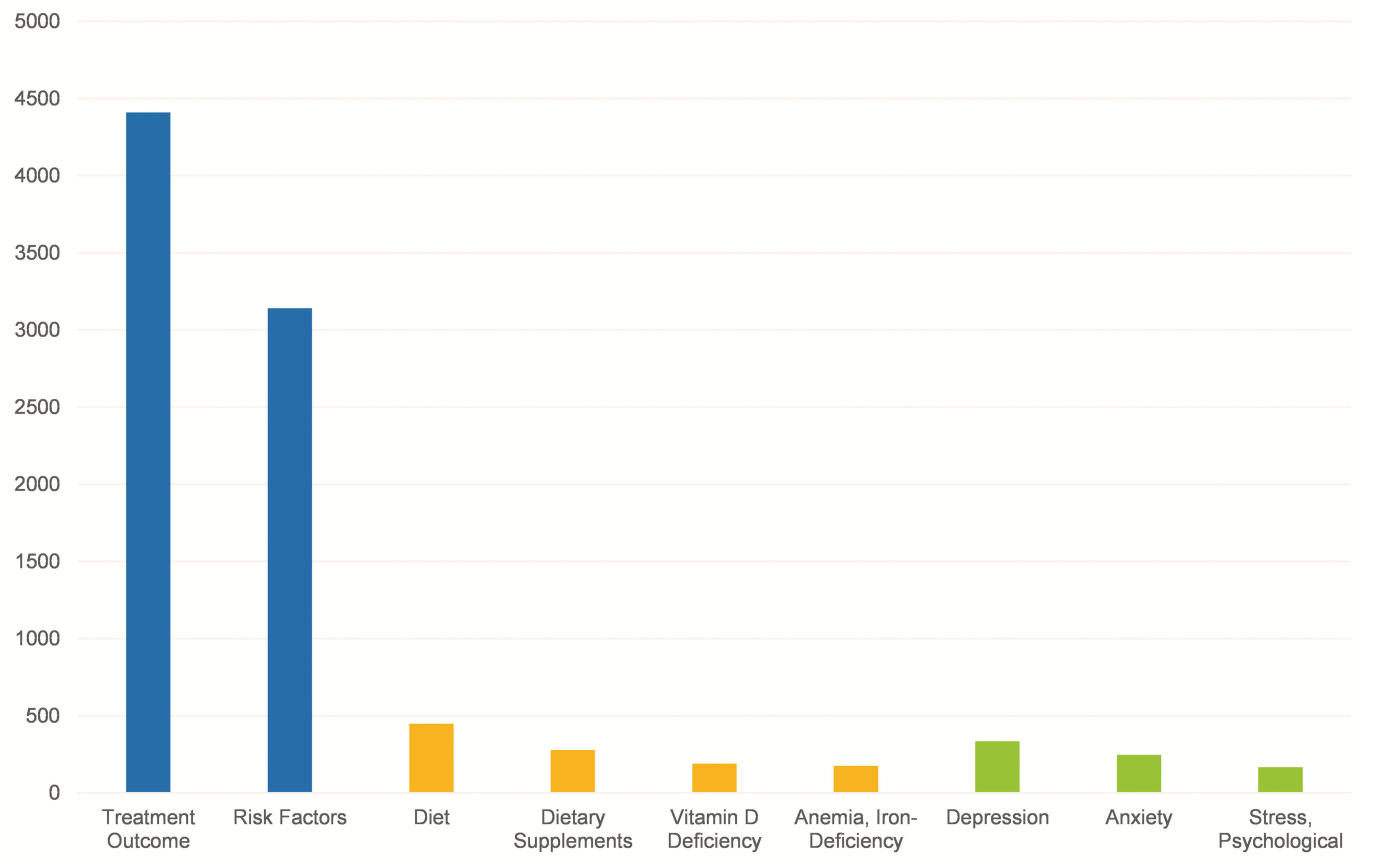
Comparison between the total amount of *Treatment Outcome* and *Risk Facters*, and some MeSH terms related to nutritional status and psychological assessment.

Figure 3 reveals that there are fewer MeSH terms on assessment of nutritional status and psychological assessment, such as Diet, Dietary Supplements, V*itamin D Deficiency, Anemia, Iron-Deficiency, Depression, Anxiety* and *Stress, Psychological* compared to the large number of *Treatment Outcome* and *Risk Factors*.

## Latent Dirichlet Allocation Analysis

**Figure 4:**
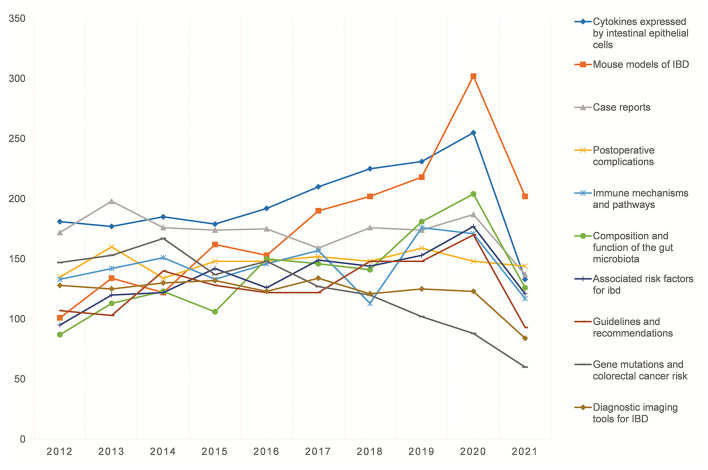
LDA analysis: the top 10 topic with the highest average number of publications per year.

LDA themes derived from publication abstracts could present more details of high-frequency research themes in the literature. Figure 4 illustrates the top 10 LDA topics with the highest average number of publications per year over the past 10 years. *Cytokines expressed by intestinal epithelial cells, Mouse Models of IBD, case reports, postoperative complications* and *immune mechanisms and pathways* are the top five LDA topics in terms of the number of publications. Moreover, *cytokines expressed by intestinal epithelial cells*, Mouse Models of IBD, *Composition and function of the gut microbiota, Guidelines and recommendations* and *Associated risk factors for IBD*-related LDA topics show an overall increasing trend during this decade; among them, *Mouse Models of IBD* and *Composition and function of the gut microbiota* increase significantly. However, *Case reports* and *Gene mutations and colorectal cancer risk* related LDA themes seem to have a decreasing trend. The remaining topics do not display large fluctuations in the number of published literatures per year.

**Figure 5:**
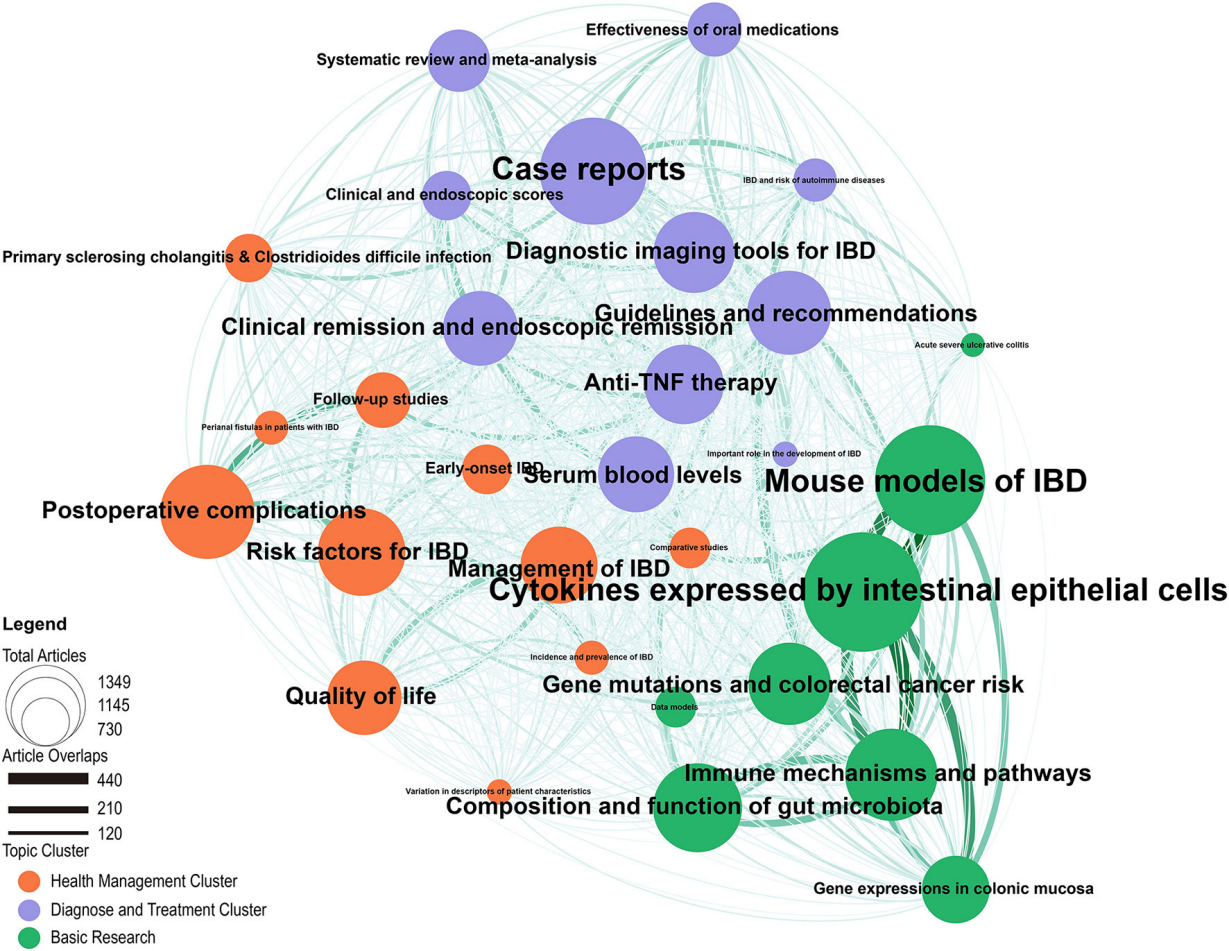
LDA research topic cluster network: inter- and intra-relationships. The orange cluster represents the “Health Management” cluster, the purple cluster represents he “Diagnose and Treatment” cluster, and the green cluster represents the “Basic Research” cluster.

Network analysis of research topics can perform clusters of topics with high similarity so that we can aggregate the interrelated topics into clusters and provide insight into the connections and strengths between different topics by investigating the network analysis. Three thematic network clusters were identified by the Louvain method (Figure 5). Publications covered in a cluster maenad exhibit a strong intracluster relationship between them. They were grouped into three fields of IBD research: *Diagnose and Treatment Cluster, Health Management Cluster* and *Basic Research*. For each theme, the size of the bubble represents the number of related papers. The lines between the bubbles indicate that the two themes use common terms, and the thickness represents the magnitude of the relationship between the themes.

In the *Diagnosis and Treatment Cluster*, the most popular topics are *Case reports, Diagnostic Imaging Tools for IBD*, Guidelines and recommendations, *Clinical remission and endoscopic remission* and *Anti-TNF therapy*. Strong relationships are seen between *Case reports, IBD and risk of autoimmune diseases* and *Effectiveness of oral medication*s. Besides, *Clinical remission* and *endoscopic remission* showed a tight association between *Anti-TNF therapy, Serum blood levels, Clinical and endoscopic scores* and *Systematic review* and *meta-analysis*.

The top three most studied topics of the *Health Management Cluster* are *Postoperative Complications, Risk factors for IBD* and *Management of IBD*. Conspicuous links can be seen among Postoperative Complications, *Risk factors for IBD, Follow-up studies* and *Perianal fistulas* in patients with IBD. *Quality of life* was strongly associated with *Management of IBD*.

The *Basic Research cluster* focuses heavily on *Cytokines expressed by intestinal epithelial cells* and *Mouse Models of IBD*. In addition, the number of papers related to the topics of *Composition and function of gut microbiota, Immune mechanisms and pathways* and *Gene mutations and colorectal cancer risk* published is close and has almost the same attraction. Noting that obvious associations are seen between *Mouse Models of IBD, Cytokines expressed by intestinal epithelial cells* and *Immune mechanisms and pathways*. Furthermore *Immune mechanisms and pathways* present tight connections with *Composition and function of gut microbiota* and *Gene expressions in colonic mucosa*. *Gene expressions in colonic mucosa* and *Cytokines expressed by intestinal epithelial cells* are also closely related. Extensive links existed between different clusters. For instance, *Case reports* connects closly with *Primary sclerosing cholangitis & Clostridioides difficile infection* and *Acute severe ulcerative colitis*. Moreover, a strong relationship was found between *Guidelines and recommendations* and *Gene mutations and colorectal cancer risk, Immune mechanisms and pathways*.

## Discussion

IBD is a chronic, incurable, inflammatory and ulcerative disease of the intestine with low mortality. It was first reported in the Western world (18), and its incidence has been steadily increasing, affecting even 0.6% of the population in Canada. Currently, the prevalence of IBD is also rapidly increasing in developing countries, suggesting that IBD is a global disease. However, for quite a long time, despite the increasing publications of IBD, the actual etiology of IBD remains elusive. Till recent years metagenomics research, high-throughput sequencing and bioinformation analysis technology has begun to emerge in medical research which contributed to the IBD research greatly. More encouragingly, in 2012, the Immunochip project of IBD led by Europe (19) found 163 genetic loci associated with IBD susceptibility, including 110 loci associated with both UC and CD, 30 loci associated only with CD, and 23 loci associated only with UC. This study provided a breakthrough understanding of genetic factors susceptible to IBD and the interactions between host and intestinal microbiomes, which offered a rich source of clues to the pathogenesis mechanisms of IBD. In short, 2012 is a milestone in the development history of IBD. Therefore, we choose 2012 as the start point and analysis the publications of the 10 years from 2012 to 2021.

Our results, showing the constantly increasing number of studies related to IBD, reflect that IBD is still receiving widespread attention. Although the disease occurs in all ages, it is mainly concentrated in adolescence and early adulthood (20), which is consistent with our result. This phenomenon leads to a complex rise in incidence and a serious burden on healthcare and the economy (21). To improve this situation, we should have a better understanding of IBD so that we can take notes on the prevention and treatment of IBD.

Our results reveal that gut flora is a hot topic in IBD-related studies, whether from the trend of MeSH terms or the ranking of LDA topics or thematic network analysis, especially in recent years, flora-related studies have witnessed growth. Together with the improvement of high-throughput sequencing technology, the role of the gut microbiome in the pathogenesis of IBD has received increasing attention, and studies on the mechanism depend on the support and validation of animal models. It is well known that intestinal flora is involved in the pathogenesis of IBD. First, the composition of the intestinal flora in IBD patients is altered. Compared with the healthy population, the diversity of intestinal flora decreases in IBD patients, while it shows an upregulation of Aspergillus and Bacteroides and a downregulation of beneficial bacteria such as Lactobacillus and Bifidobacterium (22). In a study of 40 twin pairs of IBD patients, Willing et al. found a decrease in Faecalibacterium and Roseburia but an increase in Enterobacteriaceae and Ruminococcus gnavus in patients with ilea CD (23). Second, the existence of intestinal flora is necessary for the development of IBD. In mouse model studies, the incidence and severity of intestinal inflammation were significantly reduced in Il10/-, TCRα/-, and Smad3/- mice treated with antibiotics and in germ-free mice (24–26). In population studies, we found that antibiotic treatment can relieve inflammation to some degree. To further understand how the intestinal flora participates in the development of IBD, an increasing number of studies are focusing on the relationship between the gut microbiome and the host. On the one hand, gut flora can influence the host directly; for example, bacteria are recognized by pattern recognition receptors (PRRs), which identify microorganism-associated molecular patterns (MAMPs) and trigger changes in downstream molecules and their function (27). On the other hand, the intestinal flora can also act indirectly on the host via a wide variety of metabolites. The raw materials for these metabolic processes can originate from the host or from the microbiome. Metabolites influence the host through intermediate or end products produced by intestinal flora metabolism and then participate in signal transduction. This ultimately affects the immune system, energy metabolism and mucosal barrier of the organism and participates in the development of IBD (28, 29). The metabolites induced by intestinal flora, including short-chain fatty acids (SCAFs) and bile acids, have been extensively studied. SCFAs, which are produced by flora through metabolism of carbohydrates in the intestine, have been shown to enhance regulatory T cells in the colonic mucosa, promote recovery of immune tolerance, and protect against colonic inflammation and colon cancer (30). Farnesoid X receptor (FXR), a type of bile salt, belongs to the nuclear receptor family. FXR activation inhibits the production of intestinal inflammatory factors and maintains the integrity of the intestinal epithelial barrier (31). Despite the variety of research on the mechanisms of intestinal flora in the development of IBD, it is still inconclusive whether intestinal flora and IBD are directly causal or just associated (32). However, it is undeniable that microbial-based therapy will play an essential role in the future, regardless of whether it provides theoretical support for providing flora transplantation therapy and novel methods for predicting different treatment responses of patients based on flora in the future.

IBD is an immune-mediated disease that is currently treated with 5-aminosalicylic acid, corticosteroids, immunosuppressants, and biological agents (including monoclonal antibody inhibitors of tumor necrosis factor (TNF) alpha and interleukin 12/23, integrins and small molecules such as Janus kinase (JAK) inhibitors.). The aims of the treatments are inducing or maintaining clinical and endoscopic remission and stability. Surgical-surgical interventions are considered when uncontrolled inflammation or severe complications occur. In the analysis of the treatment-related topic Mesh, we found that biologics-related subject headings, among the treatment-related topic Mesh as tumor necrosis factor inhibitors, infliximab (IFX), and adalimumab (ADA) have occupied a place in the top 10 for the last decade. IFX, the earliest anti-TNF-α monoclonal antibody, is a mouse-derived sequence chimeric with a human-derived sequence, followed by the fully humanized monoclonal antibody adalimumab (ADA) and other monoclonal antibodies. Current studies suggest that IFX is effective in inducing remission of CD and maintaining therapeutic effects for a long time (33). In a five-year study, IFX treatment did not increase the incidence of tumors and lymphomas in CD patients but increased the risk of serious infections. ADA has been recognized to induce remission in CD patients who failed IFX treatment and did not upregulate the risk of infection (34–36).

It is noteworthy that coronavirus disease 2019 (COVID-19), which first broke out in Wuhan, China, in December 2019 and rapidly became a worldwide pandemic, is a severe acute respiratory syndrome caused by a novel β-coronavirus. It is currently believed that β-coronavirus causes COVID-19 by infecting lung alveolar and intestinal epithelial cells, where ACE2 is highly expressed, followed by an immune response (37). In the context of the epidemic, IBD, as a special and large group, has received special attention. This phenomenon can also be seen in our results, and the Mesh terms of COVID-19 and SARS-CoV-2 have rapidly become a hot spot of interest since 2019. A recent meta-analysis found that the risk of suffering severe COVID-19 infection in patients with IBD was not higher than that in the general population (38). However, advanced age, ≥2 comorbidities and corticosteroid use were high risk factors for severe complications (ventilator use, intensive care unit admission and death) in patients with IBD infected with COVID-19 (39), which may contribute to the global evolution of IBD (8). Making a choice of treatments for IBD patients is crucial during the COVID-19 epidemic, and until now, reports reviewed that patients treated with corticosteroids and 5-aminosalicylates are at a greater risk for adverse outcomes, whereas the use of biologic agents appears to prevent adverse outcomes in patients (38). Therefore, it is accepted that no change in treatment is needed for patients with stable disease, but surgical operations and endoscopy need to be postponed appropriately (37). Vaccines are an effective means of preventing viral infections in humans, and it is a concern of gastroenterologists whether the benefits of vaccination outweigh the disadvantages for patients with IBD. An Israeli study of 12,109 patients with IBD demonstrated that vaccine efficacy in IBD patients did not differ from that in non-IBD patients and was not affected by TNF-α inhibitors or corticosteroid therapy; meanwhile, vaccination was not associated with IBD exacerbation (40), and a single-center cohort study in Germany also supported these findings (41). However, a study of IBD patients receiving IFX or vedolizumab in adolescents concluded that their natural infections produced lower concentrations and shorter durations of SARS-CoV-2 spike protein receptor binding domain IgG antibodies compared with non-IBD patients, which may lead to an increased risk of reinfection (42). Concomitant use of immunomodulators and IFX was also found to further attenuate the serologic response to SARS-CoV-2 infection in patients, for anti-SARS-CoV-2 antibodies detected in only one-third of patients (43). However, data on vaccination in patients with IBD are limited, and the observation period is still short; more studies and observations are needed for further clarification. Overall, the international consensus published in the GUT recommends that patients with IBD should be vaccinated as soon as the opportunity arises, regardless of whether they are receiving immunotherapy (44).

Although research on IBD is broad, it has mainly concentrated on the exploration of diagnosis, treatment, and mechanisms. However, IBD, as a chronic inflammatory disease of the intestine, causes malnutrition and psychological problems that require more attention. The relevant topics of studies on the nutritional and psychological status of IBD patients in the last 10 years from our results are deficient. The incidence of malnutrition in IBD patients has been reported to be as high as 20–85%, and malnutrition is an important factor in the poor prognosis of IBD patients. Malnutrition in IBD patients mainly manifests as weight loss, anemia, decreased muscle mass, deficiency of multiple micronutrients, low bone mass and osteoporosis (45). Vitamin D, zinc and other micronutrients are now thought to be possibly related to the pathogenesis of IBD (46). Therefore, it is particularly important to pay attention to the nutritional status of patients with IBD and provide appropriate and aggressive supplementation of relevant nutrients. Intestinal inflammation could cause a neuroinflammatory response by affecting inflammatory markers in the blood (47), and neuroinflammation can cause behavioral disturbances similar to those associated with depression or anxiety (48). A survey of 200 patients with IBD found that 27% of patients had anxiety/depression, which was strongly associated with IBD-related disability (49), and in another study, fatigue was found to be associated with poor health-related quality of life, disability and depression but not with disease control (50). The limitations of this part of the study implied that we need to devote more attention to the nutritional status and psychological status of patients in future investigations and clinical therapy.

This research has several limitations. Firstly, although PubMed is one of the world's leading medical resources, it was reported to have indexed predatory journals (51). Other databases, such as web of Science, Scopus, and Embase, should be explored in future bibliometric studies. Secondly, in our analysis, reviews were also included which may affect the findings to some extent. However, reviews are not only the overview of IBD-related research but also the summary of some meta-analysis, which may contain crucial data and reflect the topics that people concerned of IBD. so that we believe it is essential to include review articles. Thirdly, because certain freshly released publications have not yet been indexed by MeSH words, they may not appear in our analysis. And our analysis was organized in November, so the publications of December were not included in our analysis. These are common limitations in published research. Furthermore, because certain freshly released publications have not yet been indexed by MeSH words, they may not appear in our analysis. These are common limitations in published research (15). Finally, artificial intelligence was used to build the LDA themes and their connections in this study, resulting in a machine-driven understanding. In order for medical practitioners to give more effective therapy, a deeper, more complete investigation of these subjects can provide easier interpretation and more accurate outcomes.

## Conclusion

IBD is a worldwide disease whose prevalence is still on the rise globally, and the publication of literature related to IBD has been on an upward trend in the last decade. The predominant publications currently centered on IBD but restricted studies on the nutritional and psychological concerns regarding IBD, which demand more in-depth attention. In addition, it is remarkable that the management of IBD patients needs special concern when there is an outbreak or pandemic phase of some infectious disease.

## Data Availability Statement

The raw data supporting the conclusions of this article will be made available by the authors, without undue reservation.

## Author Contributions

KL, CF, YF, and JL were involved in the overall design of the study. KL, CF, and HC were responsible for acquisition, analysis, interpretation of the data, and statistical analysis. KL and CF wrote the manuscript. YF and JL assessed and interpreted the results. All authors have read and approved the published version of the manuscript.

## Funding

This work was funded by the Natural Science Foundation of China (Grant Nos. 81370500 and 81170364 to JL) and CAMS Initiative for Innovative Medicine (CAMS-2020-I2M-2-013 to JL).

## Conflict of Interest

The authors declare that the research was conducted in the absence of any commercial or financial relationships that could be construed as a potential conflict of interest.

## Publisher's Note

All claims expressed in this article are solely those of the authors and do not necessarily represent those of their affiliated organizations, or those of the publisher, the editors and the reviewers. Any product that may be evaluated in this article, or claim that may be made by its manufacturer, is not guaranteed or endorsed by the publisher.
