# Supplementary material for: Trends in Worldwide Research in Inflammatory Bowel Disease Over the Period 2012–2021: A Bibliometric Study
Source: Front Med (Lausanne). 2022 May 19;9:880553. doi: 10.3389/fmed.2022.880553 (PMC9160461; doi:10.3389/fmed.2022.880553)
Supplement: Supplementary file 1 [file Data_Sheet_1.DOCX]

Supplementary Material

The purpose of classifying tasks identified the topics number. It is difficult to present the accurate correction and compare the accuracy of different modeling due to the topic classification algorism is an unsupervised learning. However, some methods were used to explore the best topic number indeed and there are four different approaches to determine the number of the topics in our study [1-4].

In this figure, four different methods identified different numbers of topics, and there is no consistent best number of topics. Based on these four methods, we set the number of topics to 30.

**Supplementary Figure.** Performance of the four methods under different number of topics.

*Ref:*

*1. Rajkumar Arun, V. Suresh, C. E. Veni Madhavan, and M. N. Narasimha Murthy. 2010. On finding the natural number of topics with latent dirichlet allocation: Some observations. In Advances in knowledge discovery and data mining, Mohammed J. Zaki, Jeffrey Xu Yu, Balaraman Ravindran and Vikram Pudi (eds.). Springer Berlin Heidelberg, 391–402.*

*2. Cao Juan, Xia Tian, Li Jintao, Zhang Yongdong, and Tang Sheng. 2009. A density-based method for adaptive lda model selection. Neurocomputing — 16th European Symposium on Artificial Neural Networks 2008 72, 7–9: 1775–1781.*

*3. Romain Deveaud, Éric SanJuan, and Patrice Bellot. 2014. Accurate and effective latent concept modeling for ad hoc information retrieval. Document numérique 17, 1: 61–84.*

*4. Thomas L. Griffiths and Mark Steyvers. 2004. Finding scientific topics. Proceedings of the National Academy of Sciences 101, suppl 1: 5228–5235.*
